# Enhanced Electromagnetic Wave Absorption Properties of Ultrathin MnO_2_ Nanosheet-Decorated Spherical Flower-Shaped Carbonyl Iron Powder

**DOI:** 10.3390/molecules27010135

**Published:** 2021-12-27

**Authors:** Zhengwei Qu, Yi Wang, Pingan Yang, Wei Zheng, Nan Li, Jingying Bai, Youwei Zhang, Kailin Li, Dashuang Wang, Zhaohui Liu, Kexin Yao, Rui Li, Yuxin Zhang

**Affiliations:** 1 School of Automation, Chongqing University of Posts and Telecommunications, Chongqing 400065, China; laughing1203@163.com (Z.Q.); lirui@cqupt.edu.cn (R.L.); 2 College of Material Science and Engineering, Chongqing University, Chongqing 400044, China; yiwang_96@163.com (Y.W.); likailin920809@163.com (K.L.); waloneds@sina.com (D.W.); 3 Institute of Space Antenna, China Academy of Space Technology (Xi’an), Xi’an 710100, China; zhidaoyuan@163.com; 4 Aerospace Institute of Advanced Materials & Processing Technology, Beijing 100074, China; Jim1568@gmail.com; 5 Beijing Spacecraft, China Academy of Space Technology, Beijing 100194, China; baijy1213@163.com (J.B.); zhangyouwei29@163.com (Y.Z.); 6 Multi-Scale Porous Materials Center, Institute of Advanced Interdisciplinary Studies & School of Chemistry and Chemical Engineering, Chongqing University, Chongqing 400045, China; zhaohui.liu@cqu.edu.cn (Z.L.); kexinyao@cqu.edu.cn (K.Y.)

**Keywords:** spherical flower-like structure, CIP@MnO_2_, microwave absorption, interfacial polarization

## Abstract

In this work, spherical flower-shaped composite carbonyl iron powder@MnO_2_ (CIP@MnO_2_) with CIP as the core and ultrathin MnO_2_ nanosheets as the shell was successfully prepared by a simple redox reaction to improve oxidation resistance and electromagnetic wave absorption properties. The microwave-absorbing properties of CIP@MnO_2_ composites with different filling ratios (mass fractions of 20%, 40%, and 60% after mixing with paraffin) were tested and analyzed. The experimental results show that compared with pure CIP, the CIP@MnO_2_ composites have smaller minimum reflection loss and a wider effective absorption bandwidth than CIP (RL < −20 dB). The sample filled with 40 wt% has the best comprehensive performance, the minimum reflection loss is −63.87 dB at 6.32 GHz, and the effective absorption bandwidth (RL < −20 dB) reaches 7.28 GHz in the range of 5.92 GHz–9.28 GHz and 11.2 GHz–15.12 GHz, which covers most C and X bands. Such excellent microwave absorption performance of the spherical flower-like CIP@MnO_2_ composites is attributed to the combined effect of multiple beneficial components and the electromagnetic attenuation ability generated by the special spherical flower-like structure. Furthermore, this spherical flower-like core–shell shape aids in the creation of discontinuous networks, which improve microwave incidence dispersion, polarize more interfacial charges, and allow electromagnetic wave absorption. In theory, this research could lead to a simple and efficient process for producing spherical flower-shaped CIP@MnO_2_ composites with high absorption, a wide band, and oxidation resistance for a wide range of applications.

## 1. Introduction

With the rapid development of modern electronic information technology, various electronic devices have provided a lot of convenience to people while also creating potential hazards to human health [1,2]. To cope with the increasingly serious electromagnetic pollution problem, there is an urgent need to develop wave-absorbing materials with high efficiency, a wide band, and strong absorption [3,4]. CIP, as a typical magnetic loss-type radar wave absorber, has the advantages of a high specific saturation magnetization intensity, low cost, and high temperature stability. However, CIP has disadvantages, such as poor absorption performance and easy oxidation, which limit its application to real life. Some studies have shown that the absorption performance of electromagnetic wave absorbers is strongly related to the shape and structure of the material [5,6]. A larger specific surface area and a higher magnetization intensity can improve the microwave absorption performance of the material [7]. Researchers have focused on various structures to enhance microwave absorption performance. Due to high anisotropy and saturation magnetization strength, metallic magnetic particles with a flower-like morphology are of increasing interest [8]. Wang et al. synthesized flower-like nickel microcrystals by the hydrothermal method [9]. Dai et al. used a simple two-step hydrothermal method to closely mount uniform MoS_2_ nanosheets on the smooth surface of Bi_2_Fe_4_O_9_ microplatelets to form graded, structured flower-like MoS_2_@Bi_2_Fe_4_O_9_. The test results showed that the metal magnetic material with a flower-like structure has better microwave absorption performance in the GHz range [10]. Yu et al. investigated the microwave absorption performance of flower-like CIP with a minimum reflection loss (RL_min_) of −35.5 dB at 8.99 GHz and an effective absorption bandwidth (RL < −20 dB) of 3.4 GHz (7.46 GHz–10.86 GHz) [11]. However, the disadvantages of flower-like CIP, such as a narrow effective absorption bandwidth and easy oxidation, cannot meet the requirements of wave-absorbing materials for practical engineering. Therefore, various approaches have been used to prepare carbonyl iron composites with excellent electromagnetic wave absorption properties. Using different types of material cladding and combining the advantages of multiple materials are an effective method to enhance the wave absorption performance.

From the previous literature, it can be found that the effective combination of an electrically lossy material and a magnetic composite can achieve stronger attenuation performance and better impedance matching, which will result in a wider bandwidth of the composite [12]. MnO_2_ has been widely studied as a typical electro-depletion-type material with good dielectric properties and chemical stability, such as molecular adsorption, good thermal stability, easy synthesis, and environmental friendliness [13,14,15,16,17]. He et al. successfully synthesized MnO_2_ microspheres using the template-free method, and the minimum reflection loss at 14.60 GHz reached −31.79 dB and the effective absorption bandwidth below −10 dB was 5.78 GHz [18]. Liu et al. prepared MnO_2_/activated carbon composite wave-absorbing materials by the precipitation method. MnO_2_ can introduce electromagnetic waves in activated carbon and improve the wave-absorbing performance of activated carbon [19]. Chen et al. synthesized an Fe_3_O_4_@C@MnO_2_ hybrid material using simple solvothermal and redox reactions, which has good electromagnetic wave absorption with a minimum reflection loss value of −35 dB and an effective absorption bandwidth (RL < −10 dB) of 5 GHz when the thickness is 2.7 mm [20].

Based on the above considerations, the design of new flower-like core–shell structure CIP@MnO_2_ composites can improve the stability and oxidation resistance of CIP as well as enhance the wave absorption properties. In this paper, based on the redox reaction, MnO_2_ nanosheets were grown on the surface of CIP to make a composite core–shell structure with carbonyl iron as the core and MnO_2_ nanosheets as the shell. This structure enables the composite to have both high saturation and magnetization strength of the ferromagnetic metal and good dielectric properties and chemical stability at the same time. The core–shell structure can use the properties of both core/shell materials and enhance the wave-absorbing properties with the help of the flower-like structure. The difference in testing methods plays a crucial role in the accuracy of the results [21]. In this paper, the effective permittivity and permeability of the composites were obtained by the coaxial test method, and the wave absorption properties of CIP@MnO_2_ composites with mass filling fractions of 20 wt%, 40 wt%, and 60 wt% were accurately calculated. When the filling fraction is 40 wt%, composites have better integrated microwave absorption performance. This work provides some implications for the development of wave-absorbing materials with high efficiency, a broad band, and strong absorption.

## 2. Materials and Methods

### 2.1. Materials

CIP was produced by BASF AG with a particle size distribution of d50 = 3–4 μm. Potassium permanganate (KMnO_4_, ≥99%) was produced by Aladdin Co., Ltd. (Shanghai, China). Deionized water (18 MΩ cm) used to configure the solutions in the experiments was taken from the ultra-pure water machine (Millipore Aquelix 5) produced by Chongqing Jinfu Technology Company (Chongqing, China). Anhydrous ethanol (C₂H₆O) was produced by Chongqing Chuandong Chemical Co., Ltd. (Chongqing, China). All chemical reagents were of analytical grade and could be used without further treatment.

### 2.2. Synthesis

**Figure 1:**
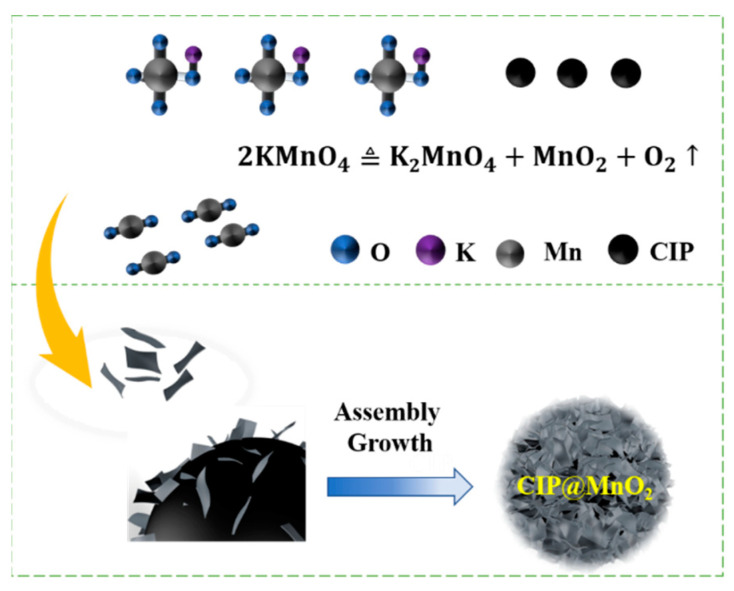
Schematic diagram of the synthesis of the CIP@MnO_2_ core–shell structure.

Figure 1 briefly illustrates the preparation process of CIP@MnO_2_ composites. In a typical process, CIP@MnO_2_ composites were synthesized by coating MnO_2_ shells on spherical carbonyl iron through a simple redox reaction. The specific steps were as follows: First, 0.5 g of CIP and 0.1106 g of KMnO_4_ were weighed and mixed in a beaker, followed by adding 70 mL of deionized water to the beaker and stirring for 20 min using mechanical stirring. Next, the stirred mixture was poured into a 100 mL PTFE-lined reactor and placed in an oven for 20 h at 160 °C and then cooled to room temperature. Finally, the CIP@MnO_2_ composite was washed with ethanol and deionized water three times and dried at 40 °C for 6 h. The CIP@MnO_2_ composite was obtained by centrifugation and drying. Taking the composite material and paraffin wax to be fully co-mingled, the composite was put into the drying oven to warm up until the paraffin wax completely melted. The melted mixture was poured into a mold with an inner diameter of 3.04 mm, an outer diameter of 7 mm, and a thickness of 3.04 mm, and a coaxial ring was formed after cooling. The coaxial line method was used to measure the electromagnetic absorption performance of the resulting samples.

### 2.3. Characterization

The morphology and structure of the spherical flower-like CIP@MnO_2_ composites were characterized using focused ion beam/scanning electron microscopy (FIB/SEM; ZEISS AURIGA, Jena, Germany) and transmission electron microscopy (TEM; FEI Talos F200S G2, Thermo Scientific, Waltham, MA, USA). X-ray powder diffraction (XRD; D/max 2500) and energy-dispersive X-ray spectroscopy (EDS) were used for determining the crystal structure and surface elemental composition. The magnetic properties of the composites were measured with a vibrating sample magnetometer (VSM, LakeShore 7404). The electromagnetic parameters were measured by a vector network analyzer (Agilent N5234A, Santa Clara, CA, USA) in the frequency range of 2–18 GHz. A reflection loss curve was drawn using the electromagnetic parameters, and the eddy current loss and impedance matching were calculated for a comprehensive analysis of the electromagnetic wave absorption performance of the CIP@MnO_2_ composite.

## 3. Results and Discussion

**Figure 2:**
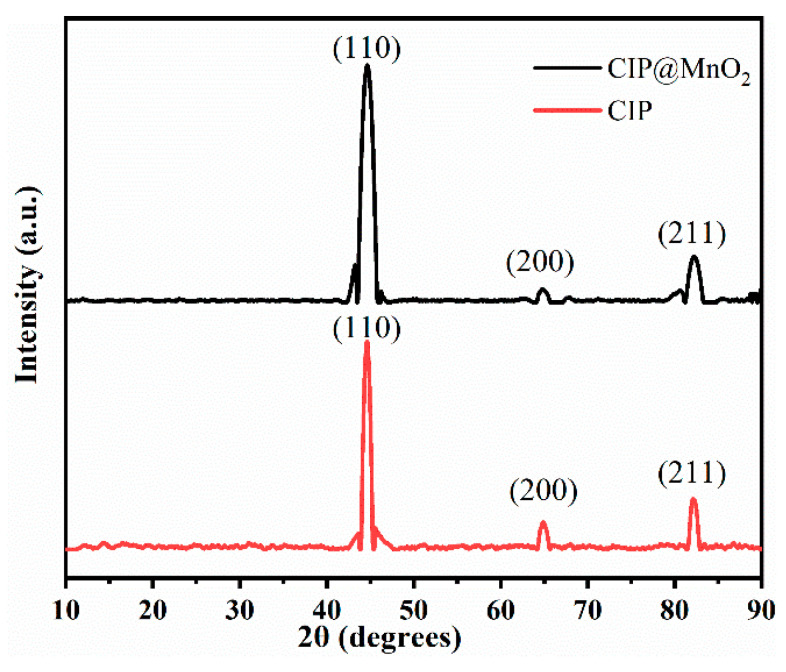
XRD spectra of CIP and CIP@MnO_2_ composites.

The structures of CIP and CIP@MnO_2_ composites were analyzed by X-ray diffraction (XRD), as shown in Figure 2. The X-ray diffractograms showed that the diffraction peaks of the pure CIP samples were located at 2θ = 44.6°, 64.9°, and 82.1°, which belong to the (110), (200), and (211) crystallographic planes of the body-centered cube, respectively. Meanwhile, the CIP@MnO_2_ composite diffraction peak was located at 2θ = 44.6°, 64.8°, and 82.2° corresponding to the (110), (200), and (211) crystal faces of the body-centered cube, respectively. However, there was no obvious characteristic peak of flake MnO_2_, which may be because the crystallinity of MnO_2_ is low, so a diffraction peak is not detected. X-ray diffraction alone was not sufficient to understand the chemical elemental composition of the CIP@MnO_2_ composites, so other means were used for an in-depth study.

**Figure 3:**
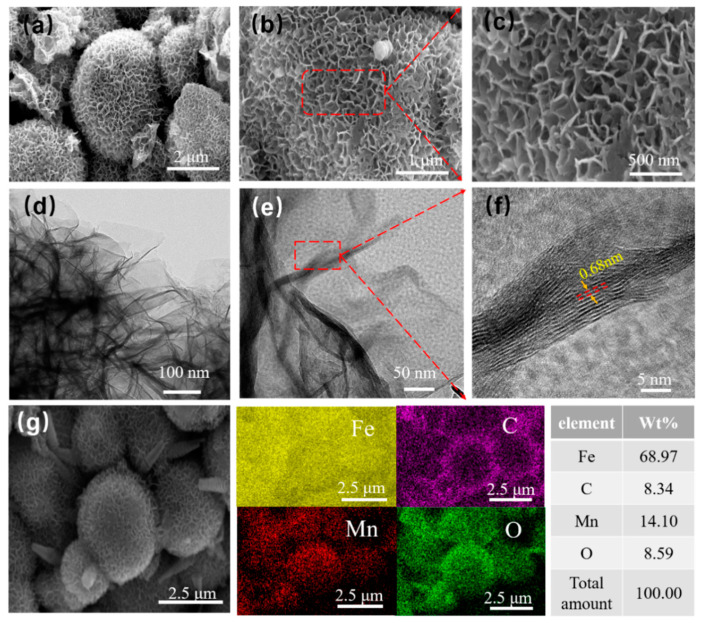
SEM images (**a**–**c**), TEM images (**d**–**f**), and element mapping (**g**) of CIP@MnO_2_ composites.

Figure 3 shows SEM images of the CIP@MnO_2_ composite samples at different magnifications. In Figure 3a, the synthesized CIP@MnO_2_ composite shows a spherical flower-like structure with agglomerates together. The CIP particles were layered and encapsulated by MnO_2_ nanosheets, which grew vertically on the surface of Fe spherical particles. Figure 3b,c shows the microscopic morphology of the same CIP@MnO_2_ composite at different magnifications under SEM, indicating that the MnO_2_ nanosheets grow vertically on the Fe sphere particles to form CIP@MnO_2_ nanospheres with a rough surface. The MnO_2_ nanosheets significantly change the surface of CIP into a spherical core-shell flower-like structure. According to Che’s theory, this irregular shape may facilitate the distribution of magnetic loss lines compared to a smooth surface [22].

To determine the microstructure and elemental distribution of the composites, limited microscopic examination and elemental distribution were performed using transmission electron microscopy (TEM) and energy-dispersive X-ray spectroscopy (EDS). As shown in Figure 3d, the MnO_2_ nanosheets were distributed on the surface of the iron sphere particles, which is consistent with the results expressed in the TEM images of the composites. Figure 3e,f shows the microstructure of the MnO_2_ nanosheets at high resolution, showing smooth folds, and the high-resolution projection images show the planar spacing of the MnO_2_ nanosheets (0.68 mm). Figure 3g shows the EDS elemental mapping images of the CIP@MnO_2_ composites, where it can be clearly observed that CIP@MnO_2_ consists of Fe, C, Mn and O elements in selected regions. C is a minority in CIP@MnO_2_. C mainly comes from carbonyl iron powder dopant Fe_3_C and free C and some adsorbed gas. The above results accurately reveal the morphology, structure, and elemental composition of the CIP@MnO_2_ composites.

**Figure 4:**
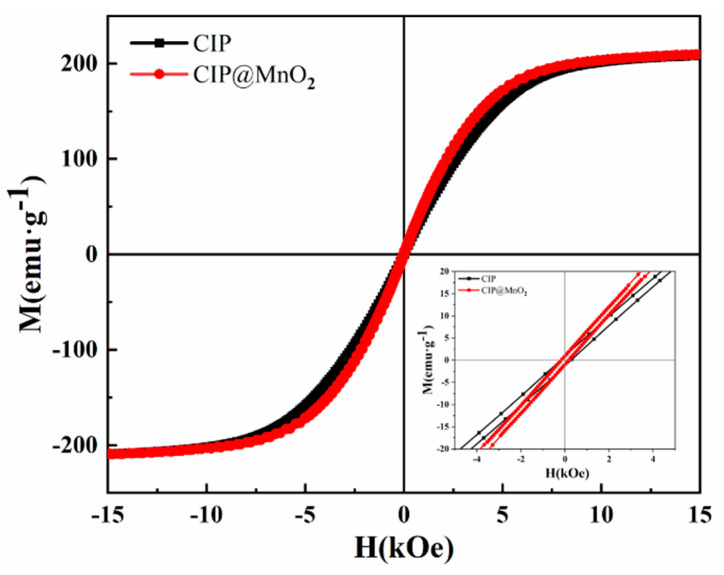
Hysteresis lines of CIP@MnO_2_ composites and CIP.

The hysteresis loops (M–H curves) of CIP@MnO_2_ composites and CIP in the range of −15 kOe to 15 kOe of the applied magnetic field were tested at 300 K, as shown in Figure 4. It can be seen that both particles exhibited typical ferromagnetic properties, i.e., the magnetization intensity of the particles varied with the increase in the applied magnetic field and then reached the saturation state. Since MnO_2_ is not magnetic, the saturation magnetization intensity of CIP@MnO_2_ is not significantly different from that of CIP. The coercivity of CIP@MnO_2_ composites (16.62 Oe) reduced compared to CIP (27.5 Oe).

It is well known that the electromagnetic wave absorption performance can be expressed by the reflection loss (RL) value [23]. The RL value can be calculated from the experimentally determined complex permittivity, characterizing the microwave absorption properties [24]. According to the transmission line theory of relative complex permittivity and magnetic permeability, the reflection loss can be expressed as [25,26,27]:

(1) $$\mathrm{RL}\left(dB\right)=20\log\left|\left(Z_{in}-Z_{0}\right)/\left(Z_{in}+Z_{0}\right)\right|$$

(2) $$Z_{in}=Z_{0}\sqrt{\mu_{r}/\varepsilon_{r}}tanh\left[j\left(2\pi fd/c\right)\sqrt{\mu_{r}\varepsilon_{r}}\right]$$

where Z*_in_* denotes the normalized input impedance of the absorbing material, *Z*_0_ denotes the input impedance in free space, $\varepsilon_{r}=\varepsilon^{\prime }-j\varepsilon^{\prime \prime }$ denotes the relative complex permittivity, and $\mu_{r}=\mu^{\prime }-j\mu^{\prime \prime }$ denotes the relative complex permeability. *f* denotes the frequency of the microwave in free space, *d* is the thickness of the absorbing material, and *c* is the propagation velocity of the electromagnetic wave in free space [28,29]. Here, the real parts of permittivity and permeability (*ε′* and *μ′*) indicate the ability to store electromagnetic energy, and the imaginary parts of permittivity and permeability (*ε″* and *μ″*) indicate the ability to lose electrical energy [30,31].

**Figure 5:**
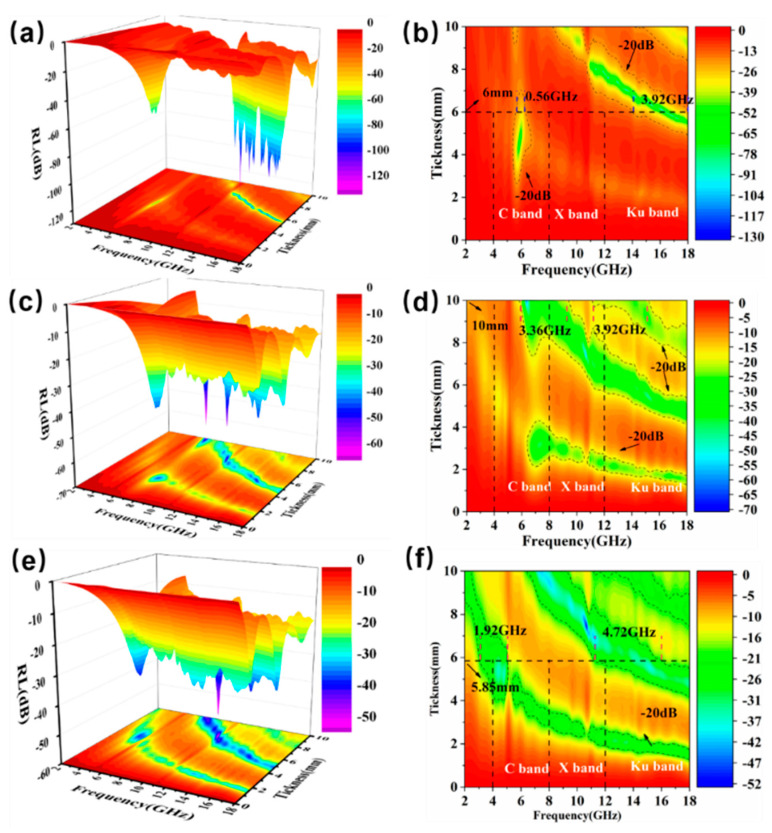
3D representation and contour plots of the reflection loss of CIP@MnO_2_ composites at different filling ratios: 20 wt% (**a**,**b**), 40 wt% (**c**,**d**), and 60 wt% (**e**,**f**).

Figure 5 shows the detailed absorption performance of the 20 wt%, 40 wt%, and 60 wt% CIP@MnO_2_ composites in the frequency range of 2–18 GHz. Figure 5b,d,f shows that the effective absorption bandwidth (RL < −20 dB) of the 20 wt%, 40 wt%, and 60 wt% CIP@MnO_2_ composites were 4.48 GHz, 7.28 GHz, and 6.64 GHz, respectively. In addition, the minimum reflection loss (RL_min_) of the samples (20 wt%, 40 wt%, and 60 wt%) reached −129.19 dB, −63.87 dB, and −52.32 dB, with matched thicknesses of 6 mm, 10 mm, and 5.85 mm, respectively. In summary, when the filling ratio is 40 wt%, the effective absorption bandwidth (RL < −20 dB) reaches 7.28 GHz and RL_min_ reaches −63.87 dB. Due to the effective absorption, the absorption bandwidth (RL < −20 dB) is wider than the filling ratio of 20 wt%, and RL_min_ is smaller than the filling ratio of 60 wt%, so the comprehensive absorption performance is better when the filling ratio is 40 wt%.

**Figure 6:**
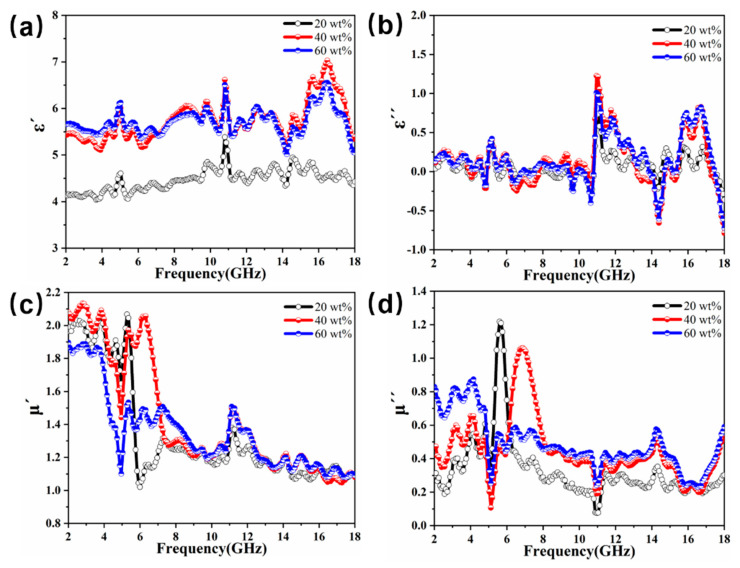
Frequency dependence of (**a**) real and (**b**) imaginary parts of complex permittivity, (**c**) real part, and (**d**) imaginary part of the relative complex permeability of the CIP@MnO_2_ composites with different filling ratios.

To further investigate the intrinsic absorption mechanism of the spherical flower-like structure CIP@MnO_2_ composites, the electromagnetic parameters and matching properties were investigated. The curves of *ε*′′ and *ε*′′ values of CIP@MnO_2_ composites with different filling ratios are depicted in Figure 6a,b, respectively. From the images, we can see that the *ε*′ values of CIP@MnO_2_ composite absorbing materials showed similar trends at different filling ratios, with an increasing trend in the tested frequency band of 2–18 GHz, and the *ε*′ values increased as the filling ratio increased. However, at fill ratios of 40–60%, *ε*′ changed relatively insignificantly, and even at higher frequency bands, *ε*′ decreased for fill ratios of 60% compared to fill ratios of 40%. There was no obvious trend of a change in *ε*′′ compared to *ε*′, the trend of a change in *ε*′′ was disorderly, and there were also some spurious peaks. There were large fluctuations in the range of 10.5–11.5 GHz and 14–45 GHz, indicating that the sample has strong absorption in the corresponding frequency band. The curves of *μ*′ and *μ*′′ values of CIP@MnO_2_ composites with different filling ratios are depicted in Figure 6b,c, from which we can see that the *μ*′ values of the three filling ratios showed a similar decreasing trend with increasing frequency. *μ*′′ showed irregular fluctuations with increasing frequency, and the fluctuation range stayed between 0.07 and 1.2.

**Figure 7:**
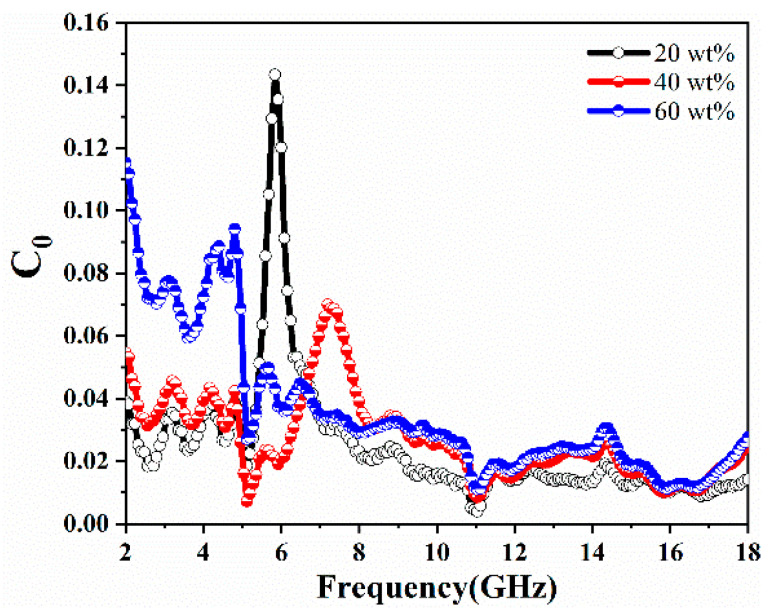
Eddy current loss (denoted by *C*_0_) with frequency for CIP@MnO_2_ composites with different filling ratios.

Usually, dielectric and magnetic losses are the main causes of electromagnetic wave attenuation [32]. Magnetic losses of metallic composites can occur at different frequency scales, including hysteresis losses, eddy current effects, domain wall resonance, and natural ferromagnetic resonance [33,34]. Hysteresis loss is negligible due to the small applied electromagnetic field [34]. The eddy current loss is related to the thickness and conductivity of the absorber and can be expressed by the following equation [35,36]:

(3) $$\mu^{\prime \prime }=2\pi \mu_{0}{\left(\mu^{\prime }\right)}^{2}\sigma d^{2}f/3$$

(4) $$C_{0}=\mu^{\prime \prime }{\left(\mu^{\prime }\right)}^{-2}f^{-1}$$

where *σ* is the electrical conductivity and *d* is the sample thickness. *μ*_0_ denotes the magnetic permeability of vacuum. Figure 7 shows the *C*_0_ values of CIP@MnO_2_ composites with different filling ratios. If the magnetic loss is caused by eddy current loss, the *C*_0_ value of the sample should be close to stable with frequency, which is not consistent with that expressed in Figure 7. So the possibility of magnetic loss caused by the eddy current effect is excluded [37]. The significant peak of the *C*_0_ curve at 4–8 GHz should be attributed to the natural resonance [2]. Due to the approximate magnetic loss properties, the variation of *C*_0_ with frequency is similar from 8 to 18 GHz [38]. Based on the above analysis, the magnetic loss in the CIP@MnO_2_ composite sample may mainly come from natural resonance.

**Figure 8:**
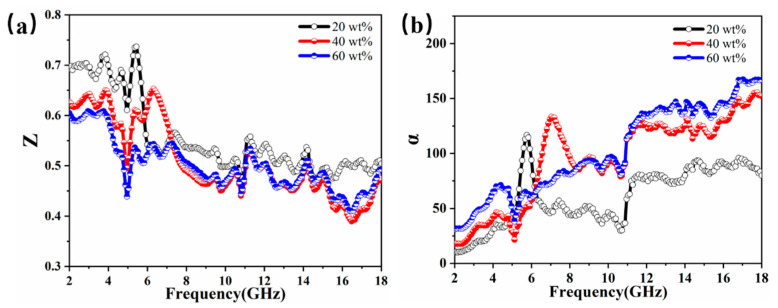
Frequency dependence of (**a**) impedance matching *Z* and (**b**) attenuation constant *α* for CIP@MnO_2_ composites with different filling ratios.

The microwave absorption performance of composite materials is the result of the combined effect of the attenuation constant and impedance matching, which is closely related to the electromagnetic properties of the sample itself. According to the principle of electromagnetic wave absorption, the impedance matching property Z is an important parameter to enhance electromagnetic wave absorption [39]. The modulus of the normalized input impedance is used to evaluate the impedance matching situation, and impedance matching can be found by the following equation [2,40,41]:

(5) $$Z=\left|\frac{Z_{in}}{Z_{0}}\right|=\left|\sqrt{\frac{\mu_{r}}{\varepsilon_{r}}}tanh\left[j\left(\frac{2\pi fd}{c}\right)\sqrt{\mu_{r}\varepsilon_{r}}\right]\right|$$

where *Z_in_* is the impedance value of the absorbing material and *Z*_0_ is the free impedance in space. When the impedance matching value is close to 1, the smaller the reflection of the incident electromagnetic wave, the ideal the impedance matching that can be achieved [42]. When the incident electromagnetic wave enters the absorbing material, it is converted into heat and other forms of energy, thus achieving the effect of absorbing electromagnetic waves [16]. Figure 8a shows the exact impedance matching values of the CIP@MnO_2_ composites, and it can be seen from the figure that the samples with different filling ratios showed a decreasing trend with increasing frequency, and the impedance matching values varied between 0.3 and 0.75, showing stronger impedance matching in the C-band.

The attenuation constant is another factor in assessing the ability of absorbing materials to attenuate microwaves and can be calculated by the following equation [43,44]:

(6) $$\alpha =\frac{\sqrt{2}\pi f}{c}\times \sqrt{\left(\mu^{\prime \prime }\varepsilon^{\prime \prime }-\mu^{\prime }\varepsilon^{\prime }\right)+\sqrt{\mu^{\prime \prime }\varepsilon^{\prime \prime }-\mu^{\prime }\varepsilon^{{}^{\prime }2}+{\left(\mu^{\prime }\varepsilon^{\prime \prime }+\mu^{\prime \prime }\varepsilon^{\prime }\right)}^{2}}}$$

where *f* is the frequency and *c* is the speed of light. The curves of the 20 wt%, 40 wt%, and 60 wt% CIP@MnO_2_ composites are shown in Figure 8b. *α* indicates the overall attenuation capability of the samples. Among the three samples, the steep peaks appearing in the 20 wt% and 40 wt% samples may be related to the strong interfacial polarization. In addition, the 60 wt% sample had the highest value, while the 20 wt% sample had the worst value.

**Figure 9:**
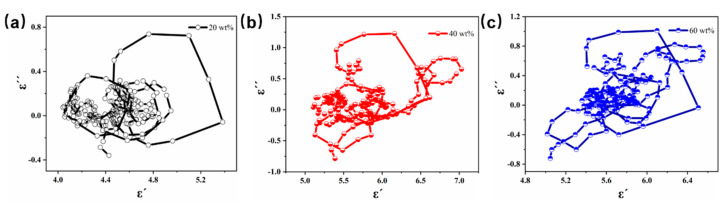
$\varepsilon^{\prime }-\varepsilon^{\prime \prime }$ plot of CIP@MnO_2_-paraffin sample with 20 wt% loading (**a**), 40 wt% loading (**b**), and 60 wt% loading (**c**).

From Debye theory, it is known that each Debye relaxation can be represented by each cole–cole semicircle in the $\varepsilon^{\prime }-\varepsilon^{\prime \prime }$ diagram. So we can use the $\varepsilon^{\prime }-\varepsilon^{\prime \prime }$ diagram to analyze the dielectric loss mechanism of composite materials [45]. Figure 9 shows the $\varepsilon^{\prime }-\varepsilon^{\prime \prime }$ plots for different fillings. From the figure, we can see that there are multiple semicircles and distorted semicircles for different filling amounts, which indicate the presence of multiple Debye relaxation and other dielectric loss mechanisms in the CIP@MnO_2_ composites [46,47].

**Table 1:** Comparison of electromagnetic wave absorption properties of CIP@MnO_2_ and its analogues.

Sample		Method	RL_min_ (dB)	EAB (GHz) (≤−20 dB)	Reference
CIP		-	−24.3	3.32	[11]
Flower-like CIP		Chemical reduction method	−35.5	3.4	[11]
CIP\MnO_2_		Mechanical ball milling	−39.1	4	[48]
MnO_2_		Hydrothermal method	−39.6	3.44	[49]
C@MnO_2_		Liquid phase method	−53.5	4.8 (<−10 dB)	[50]
CIP@MnO_2_	20 wt%	Redox reaction method	−129.19	4.48	This work
	40 wt%		−63.87	7.28	This work
	60 wt%		−52.32	6.64	This work

**Figure 10:**
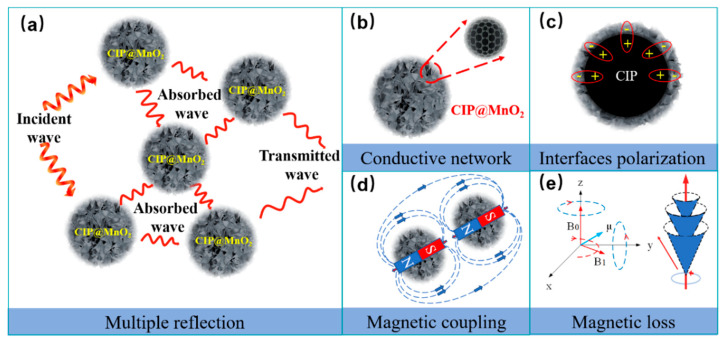
Wave absorption mechanism of CIP@MnO_2_ composites with a spherical flower-like structure: multiple reflection (**a**), conduction network (**b**), interfacial polarization (**c**), magnetic coupling (**d**), and magnetic loss (**e**).

To highlight that the CIP@MnO_2_ composite has better microwave absorption properties, Table 1 summarizes the electromagnetic wave absorption properties of some reported iron-absorbing materials (e.g., pure CIP, flower-like carbonyl iron, carbonyl iron\MnO_2_ composite, MnO_2_, C@MnO_2_ composite). Figure 10 depicts the wave absorption mechanism of the core–shell spherical flower-like structure CIP@MnO_2_ composites. When the incident wave reaches the surface of the absorbing material, the sparse and random MnO_2_ nanosheet shells facilitate the entry of electromagnetic waves. Structurally, the flower-like structure with a large specific surface area causes multiple reflections and scattering of microwaves and prolongs the transmission path, thus enhancing the absorption of electromagnetic waves [51]. However, the core–shell structure effectively increases the propagation path of microwaves and also facilitates the attenuation of electromagnetic waves [4]. In addition, the synergistic electron conduction effect of the small amount of C in CIP provides a rich conductive circuit in which the effective conduction losses can be converted from electromagnetic energy to thermal energy and disappear through electrical energy [52]. In brief, the spherical flower-like core–shell structure polarizes more interfacial charges and increases anisotropy, and electromagnetic attenuation is caused by the composite material’s intrinsic resonance.

## 4. Conclusions

A simple redox reaction was used to successfully develop MnO_2_ nanosheets on the surface of CIP to produce a spherical flower-like structure CIP@MnO_2_ composite in this research. The results show that CIP@MnO_2_ composites have stronger dielectric loss capability and exhibit better electromagnetic wave absorption performance compared with CIP itself. The effective absorption bandwidth (RL < −20 dB) is 7.28 GHz (5.92 GHz–9.28 GHz, 11.2 GHz–15.12 GHz) at a frequency of 6.32 GHz with an RL_min_ of −63.87 dB and a thickness of 10 mm when the filling amount is 40 wt%. The excellent microwave absorption performance of the spherical flower-like structure CIP@MnO_2_ composite is attributed to the combined effect of multiple beneficial components and the electromagnetic attenuation capability produced by the special spherical flower-like structure. In addition, this spherical flower-like core–shell structure contributes to the formation of a discontinuous network that enhances the dispersion of microwave incidence and polarizes more interfacial charges, which facilitate the absorption of electromagnetic waves. This study demonstrates a simple and efficient process for producing CIP@MnO_2_ microwave-absorbing materials, which exhibit high absorption, bandwidth, and oxidation resistance, as well as a wide range of practical applications.

## Data Availability

Data of the compounds are available from the authors.

## Sample Availability

Samples of the compounds are available from the authors.
